# Supplementary material for: Congruent Validity of Resting Energy Expenditure Predictive Equations in Young Adults
Source: Nutrients. 2019 Jan 22;11(2):223. doi: 10.3390/nu11020223 (PMC6413219; doi:10.3390/nu11020223)
Supplement: Supplementary file 1 [file nutrients-11-00223-s001.zip › Table S1.docx]

Table S1: Resting energy expenditure predictive equations.

| **Reference** | **Participants** | **Statistics and cross-validation** | **REE predictive equations** |
| --- | --- | --- | --- |
| Harris & Benedict (1919) | N=239 (136M, 103F), 21-70 y, 25-124.9 kg, 150-200 cm | M: r = 0.86. CL =211  F: r = 0.77. CL = 212 | M: WT*13.7516+HTCM*5.0033 –AGE*6.755+66.473  F: WT*9.5634+HTCM*1.8496-AGE*4.6756+655.0955 |
| Roza et al.  (1984) | N=337 (168M, 169F), 21-70 years, 25-124.9 kg, 150-200 cm | M: r = 0.86. CL = 213  F: r = 0.83. CL = 201 | M: 13.397*WT+4.799*HTCM–5.677*AGE+88.362  F: 9.247*WT+3.098*HTCM–4.33*AGE+477.593 |
| Bernstein et al. (1983) | N=202 (48 M, 154 F); 28-52 y, 60-204 kg, 157-182cm, BMI>30 | M: R^2^=0.449  F: R^2^=0.657  R^2^=0.485 | M: 11.02*WT+10.23*HTCM-5.8*AGE-1032  F: 7.48*WT-0.42*HTCM-3*AGE+844  19.02*FFM+3.72*FM-1.55*AGE+236.7 |
| Owen et al.  (1986) | N=104 (60 M, 44 F), 18–82 y, 60-171 kg (M) 43-153 kg (F), BMI 18–50 | M: R^2^=0.71  F: R^2^=0.74  M: R^2^=0.74  F: R^2^=0.71 | M: WT*10.2+879  F: WT*7.18+795  M: 22.3*FFM+290  F: 19.7*FFM+334 |
| Mifflin et al.  (1990) | N=498 (251 M, 248 F), N=264 normal weight (129 M, 135 F), N=234 obese (122 M, 112 F), 19–78 y, BMI 17–42 | R^2^= 0.71  R^2^= 0.64 | 9.99*WT+6.25*HTCM-4.92*AGE+166*SEX–161  19.7*FFM+413 |
| Livingston & Kohlstadt  (2005) | N=655 (299 M, 356 F), 18–95 y, 33–  278 kg | M: R^2^= 0.77  F: R^2^= 0.71 | M: 293*WT^0.4330^– 5.92*AGE  F: 248*WT^0.4356^–5.09* AGE |
|  |  |  |  |
| Schofield et al.  (1985) | N=7173, N=4814>18 y, BMI 21–24  N= 3388 Italians (47%), N=615 tropical residents, N= 322 Indian  114 published studies, N=7173 subjects (11 000 values, includes group mean values); most European and North American subjects | *r* = 0.65. SE = 0.64; *n* = 2879  *r* = 0.73. SE = 0.49; *n* = 829  *r* = 0.65. SE = 0.64; *n* = 2879  *r* = 0.73. SE = 0.49; *n* = 829 | M: AGE 18–30 y: 0.063*WT+2.896  F: AGE 18–30 y: 0.062*WT+2.036  M: AGE 18–30 y: 0.063*WT–0.042*HTM+2.953  F: AGE 18–30 y: 0.057*WT+1.148*HTM+0.411 |
| FAO  (1985) | Equation based on Schofield et al (1985);  database extended to 11 000 subjects | *r* = 0.65. SD = 151  *r* = 0.72. SD = 121  *r* = 0.65. RSD = 151  *r* = 0.73. RSD = 120 | M: AGE 18–30 y: 15.3*WT+679  F: AGE 18–30 y: 14.7*WT+496  M: AGE 18–30 y: 15.4*WT–27*HTM+717  F: AGE 18–30 y: 13.3*WT+334*HTM+35 |
| Henry et al.  (2005) | N=10552 (5794 M, 4702 F) | *r* = 0.760. SE = 0.652; *n* =2821  *r* = 0.700. SE = 0.564; *n* =1664  *r* = 0.764. SE = 0.645; *n* =2816  *r* = 0.724. SE = 0.542; *n* =1655 | M: AGE 18–30 y: 0.0669*WT+2.28  F: AGE 18–30 y: 0.0546*WT+2.33  M: AGE 18–30 y: 0.06*WT+1.31*HTM+0.473  F: AGE 18–30 y: 0.0433*WT+2.5* HTM+1.18 |
| Muller et al.  (2004) | N=2528 (1027 M, 1501 F), 5–80 y; BMI >25 | r=0.83 | 0.047*WT– 0.01452*AGE+1.009*SEX 3.21 |
|  |  | r=0.79 | BMI 25–30: 0.04507*WT-0.01553*AGE+1.006*SEX+3.407 |
|  |  | r=0.84 | BMI >30: 0.05*WT-0.01586*AGE+1.103*SEX+2.924 |
|  |  | r=0.83 | 0.05192*FFM+0.04036*FM+0.869*SEX-0.01181*AGE+2.992 |
|  |  | r=0.79 | BMI 25–30: 0.03776*FFM+0.03013*FM+0.93*SEX-0.01196*AGE+3.928 |
|  |  | r=0.84 | BMI >30: 0.05685*FFM+0.04022*FM+0.808*SEX-0.01402*AGE+2.818 |
| Korth et al.  (2007) | N=104 (50 M, 54 F), 21–68 y, BMI 18-41 | *r* = 0.84. *R*^2^= 0.71. SE = 788 | 41.5*WT+35.0*HTCM+1107.4*SEX-19.1*AGE-1731.2 |
|  |  | *r* = 0.86. *R*^2^= 0.74. SE = 732 | 108.1*FFM+1231 |
| De Lorenzo et al. (2001) | N=320 (127 M, 193 F), 18–59 y, BMI 17–40 | M: R^2^=0.597. SE=650 | M: 53.284*WT+20.957*HTCM–23.859*AGE+487 |
|  |  | F: R^2^=0.597. SE=581 | F: 46.322*WT+15.744*HTCM–16.66*AGE+944 |
| Lazzer et al.  (2007) | N= 346 (164 M, 182 F), 20–65 y, mean BMI 45 (50% FM) | M: R^2^=0.68. SE=1.14 | M: 0.048*WT+4.655*HTM-0.020*AGE-3.605 |
|  |  | F: R^2^=0.66. SE=0.56 | F: 0.042*WT+3.619*HTM-2.678 |
| Johnstone et al.  (2006) | N=150 (43 M, 107 F), 21–64 y, BMI 17–49 | R^2^=0.774 | 90.2*FFM+31.6*FM-12.2*AGE+1613 |
| Weijs & Vansant (2010) | N=536 F, >19 y, >28 BMI | R^2^ = 0.69. SEE = 204 | WT*14.038+HTCM*4.498+SEX*137.566−AGE*0.977−221.631 |
| Frankenfield  (2015) | N=337, >18 y | R^2^=0.84 | Obese: WT*10−AGE*5+SEX*274+865  Non-obese: WT*11-AGE*6+SEX*230+838  Obese: WT*10+HTCM*3−AGE*5+SEX*244+440  Non-obese: WT*10+HTCM*3−AGE*5+SEX*207+454 |
| De la Cruz et al.  (2014) | N=134 (67 M, 67 F), 19-65y | R^2^=0.68 | 1376.4–308SEX***+11.1*WT–8*AGE |
| Willis et al.  (2015) | N=159, 18-30y, 30.7 BMI mean | R^2^=0.77 | 11.2*WT−7.2*AGE+237.6*SEX+780.3 |
| De Luis et al.  (2006) | N=200 (60 M, 140 F), >20y, >30 BMI. | M: R^2^=0.70  F: R^2^=0.70 | M: 58.6+(6.1*WT)+(1023.7*HTM)–(9.5*AGE)  F: 1272.5+(9.8*WT)–(61.6*HTM)–(8.2*AGE) |

Abbreviations: M, male; F, female; y, years old; kg, kilograms; cm, centimeters; BMI, body mass index; WT, weight; HTCM, height in centimeters; FFM, fat free mass; FM, fat mass; HTM, height in meters; ***Female*1, male*0.
