# Supplementary material for: Congruent Validity of Resting Energy Expenditure Predictive Equations in Young Adults
Source: Nutrients. 2019 Jan 22;11(2):223. doi: 10.3390/nu11020223 (PMC6413219; doi:10.3390/nu11020223)
Supplement: Supplementary file 1 [file nutrients-11-00223-s001.zip › Table S2.docx]

Table S2a: Validity of resting energy expenditure (REE) predictive equations in normal-weight young men.

| **REE predictive equation** | **N** | **^1^REE**  **(Kcal/day)** | **P value ANCOVA^2^** | **Mean BIAS^3^ (Kcal/day)** | **Lower limit of agreement (Kcal/day)** | **Higher limit of agreement (Kcal/day)** | **Mean absolute differences^4^ (Kcal/day)** | **Percentage of accurate predictions (10%)^5^** | **Percentage of under predictions (10%)^6^** | **Percentage of over predictions (10%)^7^** | **Percentage of accurate predictions (5%)^8^** | **Percentage of under predictions (5%)^9^** | **Percentage of over predictions (5%)^10^** |
| --- | --- | --- | --- | --- | --- | --- | --- | --- | --- | --- | --- | --- | --- |
| Harris & Benedict (1) | 20 | 1746±124 | 0.248 | -160 | -845 | 526 | 286±241 | 45 | 10 | 45 | 30 | 20 | 50 |
| Roza et al. (2) | 20 | 1731±121 | 0.253 | -145 | -830 | 541 | 281±237 | 45 | 10 | 45 | 30 | 20 | 50 |
| Bernstein et al. (3)a | 20 | 1397±129 | 0.250 | 190 | -481 | 862 | 303±231 | 25 | 55 | 20 | 5 | 60 | 35 |
| Bernstein et al. (3)b | 20 | 1220±104 | 0.244 | 367 | -320 | 1054 | 396±306 | 20 | 70 | 10 | 15 | 75 | 10 |
| Owen et al. (4)a | 20 | 1582±77 | 0.317 | 5 | -710 | 719 | 282±208 | 30 | 30 | 40 | 10 | 50 | 40 |
| Owen et al. (4)b | 20 | 1406±119 | 0.226 | 180 | -500 | 861 | 297±239 | 25 | 50 | 25 | 15 | 60 | 25 |
| Mifflin et al. (5)a | 20 | 1684±102 | 0.277 | -97 | -788 | 594 | 271±226 | 45 | 15 | 40 | 30 | 25 | 45 |
| Mifflin et al. (5)b | 20 | 1399±105 | 0.245 | 188 | -501 | 876 | 302±243 | 25 | 50 | 25 | 15 | 60 | 25 |
| Livingston et al. (6) | 20 | 1702±87 | 0.283 | -115 | -833 | 602 | 281±243 | 45 | 10 | 45 | 30 | 25 | 45 |
| Schofield et al. (7)a | 20 | 1729±114 | 0.274 | -142 | -836 | 551 | 281±240 | 45 | 10 | 45 | 35 | 15 | 50 |
| Schofield et al. (7)b | 20 | 1725±113 | 0.274 | -138 | -832 | 555 | 280±239 | 45 | 10 | 45 | 35 | 15 | 50 |
| FAO (8)a | 20 | 1734±116 | 0.271 | -147 | -840 | 545 | 282±242 | 45 | 10 | 45 | 35 | 15 | 50 |
| FAO (8)a | 20 | 1731±115 | 0.272 | -145 | -838 | 549 | 282±241 | 45 | 10 | 45 | 35 | 15 | 50 |
| Henry et al. (9)a | 20 | 1646±121 | 0.266 | -60 | -750 | 631 | 273±209 | 40 | 20 | 40 | 15 | 40 | 45 |
| Henry et al. (9)b | 20 | 1650±121 | 0.265 | -63 | -744 | 618 | 270±207 | 45 | 15 | 40 | 20 | 35 | 45 |
| Muller et al. (10)a | 20 | 1613±203 | 0.827 | -26 | -874 | 822 | 310±281 | 35 | 25 | 40 | 20 | 35 | 45 |
| Muller et al. (10)b | 20 | 1535±212 | 0.816 | 52 | -763 | 866 | 305±266 | 35 | 30 | 35 | 10 | 45 | 45 |
| Korth et al. (11)a | 20 | 1902±112 | 0.270 | -315 | -996 | 366 | 381±259 | 30 | 5 | 65 | 15 | 5 | 80 |
| Korth et al. (11)b | 20 | 1587±138 | 0.202 | 0 | -672 | 672 | 266±194 | 35 | 30 | 35 | 15 | 45 | 40 |
| De Lorenzo et al. (12) | 20 | 1749±117 | 0.258 | -162 | -849 | 525 | 286±243 | 45 | 10 | 45 | 30 | 20 | 50 |
| Johnstone et al. (13) | 20 | 1532±123 | 0.219 | 55 | -621 | 731 | 272±197 | 20 | 45 | 35 | 15 | 45 | 40 |
| Frankenfield (14)a | 20 | 1769±87 | 0.292 | -182 | -889 | 525 | 299±255 | 50 | 5 | 45 | 30 | 15 | 55 |
| Frankenfield (14)b | 20 | 1628±75 | 0.304 | -41 | -766 | 683 | 281±222 | 40 | 20 | 40 | 15 | 40 | 45 |
| De la Cruz et al. (15) | 20 | 1814±240 | 0.878 | -228 | -1091 | 636 | 388±285 | 35 | 10 | 55 | 10 | 20 | 70 |
| Willis et al. (16) | 20 | 1635±84 | 0.291 | -48 | -769 | 673 | 280±222 | 40 | 20 | 40 | 15 | 40 | 45 |

^1^REE obtained by predictive equations (Mean±SD); ^2^P value of the main effect of ANCOVA comparing measured and predicted REE adjusting for the metabolic cart used; ^3^Mean error between measured value and predictive equation (measured – predicted); ^4^Mean of absolute differences between measured and predictive value (Mean±SD); ^5^Percentage of subjects predicted by this predictive equation within ±10% of the measured value; ^6^Percentage of subjects predicted by this predictive equation <10% of the measured value; ^7^Percentage of subjects predicted by this predictive equation >10% of the measured value; ^8^Percentage of subjects predicted by this predictive equation within ±10% of the measured value; ^9^Percentage of subjects predicted by this predictive equation <10% of the measured value; ^10^Percentage of subjects predicted by this predictive equation >10% of the measured value. *P<0.05, **P<0.01, ***P<0.001, ANCOVA test. (a) and (b) refer to different predictive equations which are proposed by the same author but require different anthropometry or body composition parameters.

Table S2b: Validity of resting energy expenditure (REE) predictive equations in overweight young men.

| **REE predictive equation** | **N** | **^1^REE**  **(Kcal/day)** | **P value ANCOVA^2^** | **Mean BIAS^3^ (Kcal/day)** | **Lower limit of agreement (Kcal/day)** | **Higher limit of agreement (Kcal/day)** | **Mean absolute differences^4^ (Kcal/day)** | **Percentage of accurate predictions (10%)^5^** | **Percentage of under predictions (10%)^6^** | **Percentage of over predictions (10%)^7^** | **Percentage of accurate predictions (5%)^8^** | **Percentage of under predictions (5%)^9^** | **Percentage of over predictions (5%)^10^** |
| --- | --- | --- | --- | --- | --- | --- | --- | --- | --- | --- | --- | --- | --- |
| Harris & Benedict (1) | 12 | 1950±148 | 0.199 | -276 | -1170 | 618 | 392±338 | 33.3 | 8.3 | 58.3 | 25 | 16.7 | 58.3 |
| Roza et al. (2) | 12 | 1931±143 | 0.195 | -257 | -1144 | 630 | 382±330 | 41.7 | 8.3 | 50 | 25 | 16.7 | 58.3 |
| Bernstein et al. (3)a | 12 | 1566±168 | 0.212 | 109 | -805 | 1023 | 381±251 | 25 | 41.7 | 33.3 | 8.3 | 58.3 | 33.3 |
| Bernstein et al. (3)b | 12 | 1365±77 | 0.110 | 309 | -417 | 1035 | 384±273 | 16.7 | 66.7 | 16.7 | 8.3 | 66.7 | 25 |
| Owen et al. (4)a | 12 | 1739±77 | 0.155 | -65 | -873 | 744 | 314±245 | 33.3 | 25 | 41.7 | 25 | 33.3 | 41.7 |
| Owen et al. (4)b | 12 | 1542±91 | 0.089 | 132 | -556 | 820 | 300±196 | 25 | 50 | 25 | 8.3 | 58.3 | 33.3 |
| Mifflin et al. (5)a | 12 | 1834±128 | 0.191 | -160 | -1024 | 704 | 349±284 | 33.3 | 16.7 | 50 | 25 | 25 | 50 |
| Mifflin et al. (5)b | 12 | 1519±80 | 0.093 | 155 | -535 | 845 | 307±205 | 25 | 50 | 25 | 0 | 66.7 | 33.3 |
| Livingston et al. (6) | 12 | 1858±83 | 0.168 | -184 | -998 | 631 | 342±273 | 33.3 | 16.7 | 50 | 33.3 | 16.7 | 50 |
| Schofield et al. (7)a | 12 | 1961±114 | 0.165 | -286 | -1141 | 568 | 395±318 | 33.3 | 8.3 | 58.3 | 16.7 | 16.7 | 66.7 |
| Schofield et al. (7)a | 12 | 1957±113 | 0.165 | -282 | -1136 | 572 | 392±317 | 33.3 | 8.3 | 58.3 | 16.7 | 16.7 | 66.7 |
| FAO (8)a | 12 | 1969±116 | 0.166 | -295 | -1152 | 562 | 401±320 | 33.3 | 8.3 | 58.3 | 16.7 | 16.7 | 66.7 |
| FAO (8)a | 12 | 1968±115 | 0.165 | -294 | -1150 | 562 | 400±319 | 33.3 | 8.3 | 58.3 | 16.7 | 16.7 | 66.7 |
| Henry et al. (9)a | 12 | 1892±121 | 0.167 | -218 | -1082 | 646 | 367±301 | 33.3 | 16.7 | 50 | 25 | 16.7 | 58.3 |
| Henry et al. (9)b | 12 | 1874±132 | 0.175 | -199 | -1073 | 675 | 363±300 | 33.3 | 16.7 | 50 | 25 | 16.7 | 58.3 |
| Muller et al. (10)a | 12 | 1750±149 | 0.162 | -76 | -872 | 721 | 319±231 | 33.3 | 25 | 41.7 | 25 | 33.3 | 41.7 |
| Muller et al. (10)b | 12 | 1712±161 | 0.189 | -37 | -825 | 750 | 312±224 | 33.3 | 25 | 41.7 | 25 | 33.3 | 41.7 |
| Korth et al. (11)a | 12 | 2054±144 | 0.198 | -380 | -1262 | 502 | 457±351 | 33.3 | 8.3 | 58.3 | 8.3 | 8.3 | 83.3 |
| Korth et al. (11)b | 12 | 1744±105 | 0.084 | -70 | -755 | 616 | 271±205 | 41.7 | 25 | 33.3 | 25 | 25 | 50 |
| De Lorenzo et al. (12) | 12 | 1939±139 | 0.194 | -264 | -1146 | 617 | 385±330 | 33.3 | 8.3 | 58.3 | 25 | 16.7 | 58.3 |
| Johnstone et al. (13) | 12 | 1724±94 | 0.123 | -49 | -812 | 713 | 298±226 | 41.7 | 25 | 33.3 | 16.7 | 41.7 | 41.7 |
| Frankenfield (14)a | 12 | 1916±103 | 0.178 | -242 | -1078 | 594 | 367±301 | 41.7 | 8.3 | 50 | 16.7 | 16.7 | 66.7 |
| Frankenfield (14)b | 12 | 1770±81 | 0.171 | -96 | -910 | 718 | 317±256 | 41.7 | 16.7 | 41.7 | 16.7 | 33.3 | 50 |
| De la Cruz et al. (15) | 12 | 2028±186 | 0.193 | -353 | -1175 | 468 | 444±298 | 16.7 | 16.7 | 66.7 | 8.3 | 16.7 | 75 |
| Willis et al. (16) | 12 | 1793±92 | 0.176 | -119 | -945 | 707 | 325±265 | 41.7 | 16.7 | 41.7 | 16.7 | 33.3 | 50 |

^1^REE obtained by predictive equations (Mean±SD); ^2^P value of the main effect of ANCOVA comparing measured and predicted REE adjusting for the metabolic cart used; ^3^Mean error between measured value and predictive equation (measured – predicted); ^4^Mean of absolute differences between measured and predictive value (Mean±SD); ^5^Percentage of subjects predicted by this predictive equation within ±10% of the measured value; ^6^Percentage of subjects predicted by this predictive equation <10% of the measured value; ^7^Percentage of subjects predicted by this predictive equation >10% of the measured value; ^8^Percentage of subjects predicted by this predictive equation within ±10% of the measured value; ^9^Percentage of subjects predicted by this predictive equation <10% of the measured value; ^10^Percentage of subjects predicted by this predictive equation >10% of the measured value. *P<0.05, **P<0.01, ***P<0.001, ANCOVA test. (a) and (b) refer to different predictive equations which are proposed by the same author but require different anthropometry or body composition parameters.

Table S2c: Validity of resting energy expenditure (REE) predictive equations in obese young men.

| **REE predictive equation** | **N** | **^1^REE**  **(Kcal/day)** | **P value ANCOVA^2^** | **Mean BIAS^3^ (Kcal/day)** | **Lower limit of agreement (Kcal/day)** | **Higher limit of agreement (Kcal/day)** | **Mean absolute differences^4^ (Kcal/day)** | **Percentage of accurate predictions (10%)^5^** | **Percentage of under predictions (10%)^6^** | **Percentage of over predictions (10%)^7^** | **Percentage of accurate predictions (5%)^8^** | **Percentage of under predictions (5%)^9^** | **Percentage of over predictions (5%)^10^** |
| --- | --- | --- | --- | --- | --- | --- | --- | --- | --- | --- | --- | --- | --- |
| Harris & Benedict (1) | 11 | 2298±166 | 0.115 | -428 | -816 | -40 | 427±193 | 18.2 | 0 | 81.8 | 9.1 | 0 | 90.9 |
| Roza et al. (2) | 11 | 2270±162 | 0.121 | -400 | -784 | -15 | 399±192 | 18.2 | 0 | 81.8 | 9.1 | 0 | 90.9 |
| Bernstein et al. (3)a | 11 | 1852±167 | 0.080 | 18 | -363 | 399 | 140±122 | 72.7 | 18.2 | 9.1 | 36.4 | 27.3 | 36.4 |
| Bernstein et al. (3)b | 11 | 1588±134 | 0.062 | 282 | -131 | 694 | 291±190 | 27.3 | 72.7 | 0 | 9.1 | 90.9 | 0 |
| Owen et al. (4)a | 11 | 1990±107 | 0.233 | -120 | -514 | 273 | 190±121 | 63.6 | 0 | 36.4 | 18.2 | 18.2 | 63.6 |
| Owen et al. (4)b | 11 | 1737±143 | 0.400 | 133 | -286 | 552 | 191±151 | 54.5 | 36.4 | 9.1 | 45.5 | 45.5 | 9.1 |
| Mifflin et al. (5)a | 11 | 2090±135 | 0.120 | -220 | -607 | 167 | 250±146 | 36.4 | 0 | 63.6 | 18.2 | 9.1 | 72.7 |
| Mifflin et al. (5)b | 11 | 1691±127 | 0.054 | 179 | -241 | 599 | 210±174 | 45.5 | 45.5 | 9.1 | 18.2 | 72.7 | 9.1 |
| Livingston et al. (6) | 11 | 2095±±90 | 0.209 | -225 | -647 | 197 | 264±152 | 36.4 | 0 | 63.6 | 9.1 | 9.1 | 81.8 |
| Schofield et al. (7)a | 11 | 2332±158 | 0.207 | -462 | -837 | -86 | 461±187 | 9.1 | 0 | 90.9 | 0 | 0 | 100 |
| Schofield et al. (7)a | 11 | 2327±158 | 0.208 | -457 | -833 | -82 | 457±187 | 18.2 | 0 | 81.8 | 0 | 0 | 100 |
| FAO (8)a | 11 | 2346±161 | 0.206 | -476 | -852 | -101 | 476±187 | 9.1 | 0 | 90.9 | 0 | 0 | 100 |
| FAO (8)a | 11 | 2347±161 | 0.210 | -477 | -853 | -101 | 477±187 | 9.1 | 0 | 90.9 | 0 | 0 | 100 |
| Henry et al. (9)a | 11 | 2286±168 | 0.206 | -416 | -791 | -41 | 415±187 | 18.2 | 0 | 81.8 | 9.1 | 0 | 90.9 |
| Henry et al. (9)b | 11 | 2230±166 | 0.165 | -360 | -729 | 8 | 360±184 | 27.3 | 0 | 72.7 | 9.1 | 0 | 90.9 |
| Muller et al. (10)a | 11 | 1848±434 | 0.013 | 22 | -1042 | 1085 | 481±169 | 0 | 54.5 | 45.5 | 0 | 54.5 | 45.5 |
| Muller et al. (10)b | 11 | 1803±479 | 0.016 | 67 | -1065 | 1199 | 514±186 | 0 | 54.5 | 45.5 | 0 | 54.5 | 45.5 |
| Korth et al. (11)a | 11 | 2310±146 | 0.101 | -440 | -821 | -60 | 440±190 | 18.2 | 0 | 81.8 | 9.1 | 0 | 100 |
| Korth et al. (11)b | 11 | 1969±166 | 0.027 | -99 | -522 | 323 | 200±105 | 54.5 | 9.1 | 36.4 | 18.2 | 27.3 | 54.5 |
| De Lorenzo et al. (12) | 11 | 2261±156 | 0.120 | -391 | -776 | -6 | 390±192 | 18.2 | 0 | 81.8 | 9.1 | 0 | 100 |
| Lazzer et al. (17) | 11 | 2098±384 | 0.047 | -228 | -1034 | 579 | 382±241 | 27.3 | 9.1 | 63.6 | 18.2 | 9.1 | 72.7 |
| Johnstone et al. (13) | 11 | 2027±164 | 0.054 | -157 | -568 | 253 | 222±122 | 36.4 | 0 | 63.6 | 27.3 | 9.1 | 63.6 |
| Frankenfield (14)a | 11 | 2191±117 | 0.159 | -321 | -719 | 77 | 327±186 | 27.3 | 0 | 72.7 | 18.2 | 0 | 81.8 |
| Frankenfield (14)b | 11 | 2113±102 | 0.203 | -244 | -655 | 168 | 273±159 | 36.4 | 0 | 63.6 | 18.2 | 0 | 81.8 |
| De la Cruz et al. (15) | 11 | 2019±404 | 0.013 | -149 | -1180 | 882 | 453±252 | 18.2 | 36.4 | 45.5 | 9.1 | 45.5 | 45.5 |
| Willis et al. (16) | 11 | 2298±166 | 0.182 | -428 | -816 | -40 | 246±143 | 36.4 | 0 | 63.6 | 18.2 | 9.1 | 72.7 |
| De Luis et al (18) | 11 | 2321±119 | 0.712 | -450 | -896 | -92 | 450±206 | 18.2 | 0 | 81.8 | 9.1 | 0 | 90.9 |

^1^REE obtained by predictive equations (Mean±SD); ^2^P value of the main effect of ANCOVA comparing measured and predicted REE adjusting for the metabolic cart used; ^3^Mean error between measured value and predictive equation (measured – predicted); ^4^Mean of absolute differences between measured and predictive value (Mean±SD); ^5^Percentage of subjects predicted by this predictive equation within ±10% of the measured value; ^6^Percentage of subjects predicted by this predictive equation <10% of the measured value; ^7^Percentage of subjects predicted by this predictive equation >10% of the measured value; ^8^Percentage of subjects predicted by this predictive equation within ±10% of the measured value; ^9^Percentage of subjects predicted by this predictive equation <10% of the measured value; ^10^Percentage of subjects predicted by this predictive equation >10% of the measured value. *P<0.05, **P<0.01, ***P<0.001, ANCOVA test. (a) and (b) refer to different predictive equations which are proposed by the same author but required different anthropometry or body composition parameters.

Table S2d: Validity of resting energy expenditure (REE) predictive equations in normal-weight young women.

| **REE predictive equation** | **N** | **^1^REE**  **(Kcal/day)** | **P value ANCOVA^2^** | **Mean BIAS^3^ (Kcal/day)** | **Lower limit of agreement (Kcal/day)** | **Higher limit of agreement (Kcal/day)** | **Mean absolute differences^4^ (Kcal/day)** | **Percentage of accurate predictions (10%)^5^** | **Percentage of under predictions (10%)^6^** | **Percentage of over predictions (10%)^7^** | **Percentage of accurate predictions (5%)^8^** | **Percentage of under predictions (5%)^9^** | **Percentage of over predictions (5%)^10^** |
| --- | --- | --- | --- | --- | --- | --- | --- | --- | --- | --- | --- | --- | --- |
| Harris & Benedict (1) | 59 | 1419±77 | 0.030 | -124 | -532 | 284 | 171±165 | 52.5 | 5.1 | 42.4 | 32.2 | 10.2 | 57.6 |
| Roza et al. (2) | 59 | 1435±82 | 0.028 | -141 | -550 | 268 | 179±170 | 52.5 | 3.4 | 44.1 | 27.1 | 10.2 | 62.7 |
| Bernstein et al. (3)a | 59 | 1149±50 | 0.035 | 145 | -268 | 558 | 216±128 | 23.7 | 62.7 | 13.6 | 11.9 | 74.6 | 13.6 |
| Bernstein et al. (3)b | 59 | 976±84 | 0.027 | 318 | -95 | 731 | 341±163 | 10.2 | 86.4 | 3.4 | 10.2 | 86.4 | 3.4 |
| Owen et al. (4)a | 59 | 1218±51 | 0.030 | 77 | -339 | 492 | 181±125 | 37.3 | 47.5 | 15.3 | 18.6 | 59.3 | 22 |
| Owen et al. (4)b | 59 | 1055±80 | 0.029 | 239 | -179 | 658 | 281±146 | 18.6 | 76.3 | 5.1 | 5.1 | 83.1 | 11.9 |
| Mifflin et al. (5)a | 59 | 1345±106 | 0.260 | -50 | -465 | 364 | 142±157 | 61 | 11.9 | 27.1 | 40.7 | 22 | 37.3 |
| Mifflin et al. (5)b | 59 | 1134±80 | 0.029 | 160 | -258 | 579 | 226±133 | 20.3 | 66.1 | 13.6 | 10.2 | 76.3 | 13.6 |
| Livingston et al. (6) | 59 | 1349±76 | 0.034 | -55 | -459 | 350 | 145±149 | 64.4 | 10.2 | 25.4 | 33.9 | 23.7 | 42.4 |
| Schofield et al. (7)a | 59 | 1359±105 | 0.024 | -64 | -476 | 348 | 150±153 | 64.4 | 10.2 | 25.4 | 33.9 | 22 | 44.1 |
| Schofield et al. (7)a | 59 | 1350±111 | 0.022 | -56 | -471 | 359 | 147±155 | 64.4 | 10.2 | 25.4 | 33.9 | 23.7 | 42.4 |
| FAO (8)a | 59 | 1362±104 | 0.024 | -67 | -479 | 344 | 150±154 | 62.7 | 10.2 | 27.1 | 33.9 | 22 | 44.1 |
| FAO (8)a | 59 | 1367±112 | 0.022 | -72 | -488 | 343 | 150±159 | 62.7 | 10.2 | 27.1 | 37.3 | 16.9 | 45.8 |
| Henry et al. (9)a | 59 | 1325±93 | 0.024 | -30 | -440 | 380 | 145±146 | 62.7 | 15.3 | 22 | 33.9 | 28.8 | 37.3 |
| Henry et al. (9)b | 59 | 1335±108 | 0.021 | -40 | -458 | 378 | 145±154 | 59.3 | 13.6 | 27.1 | 37.3 | 25.4 | 37.3 |
| Muller et al. (10)a | 59 | 1478±201 | 0.116 | -183 | -677 | 311 | 227±206 | 44.1 | 3.4 | 52.5 | 23.7 | 13.6 | 62.7 |
| Muller et al. (10)b | 59 | 1398±204 | 0.211 | -104 | -604 | 396 | 205±174 | 45.8 | 15.3 | 39 | 18.6 | 30.5 | 50.8 |
| Korth et al. (11)a | 59 | 1442±118 | 0.025 | -147 | -568 | 274 | 184±178 | 52.5 | 3.4 | 44.1 | 27.1 | 10.2 | 62.7 |
| Korth et al. (11)b | 59 | 1239±105 | 0.028 | 55 | -367 | 478 | 168±137 | 52.5 | 30.5 | 16.9 | 16.9 | 61 | 22 |
| De Lorenzo et al. (12) | 59 | 1407±98 | 0.026 | -112 | -523 | 298 | 163±166 | 52.5 | 6.8 | 40.7 | 37.3 | 13.6 | 49.2 |
| Johnstone et al. (13) | 59 | 1267±103 | 0.026 | 27 | -385 | 439 | 152±140 | 59.3 | 22 | 18.6 | 25.4 | 50.8 | 23.7 |
| Weijs & Vansant (18) | 59 | 1460±124 | 0.023 | -165 | -585 | 254 | 196±180 | 47.5 | 3.4 | 49.2 | 20.3 | 10.2 | 69.5 |
| Frankenfield (14)a | 59 | 1425±86 | 0.029 | -131 | -539 | 278 | 173±168 | 52.5 | 3.4 | 44.1 | 28.8 | 10.2 | 61 |
| Frankenfield (14)b | 59 | 1295±70 | 0.035 | 0 | -406 | 406 | 148±136 | 55.9 | 22 | 22 | 35.6 | 39 | 25.4 |
| De la Cruz et al. (15) | 58 | 1671±281 | 0.509 | -381 | -1003 | 240 | 385±305 | 22 | 0 | 78 | 11.9 | 1.7 | 86.4 |
| Willis et al. (16) | 59 | 1281±79 | 0.035 | 13 | -391 | 418 | 148±136 | 55.9 | 23.7 | 20.3 | 32.2 | 42.4 | 25.4 |

^1^REE obtained by predictive equations (Mean±SD); ^2^P value of the main effect of ANCOVA comparing measured and predicted REE adjusting for the metabolic cart used; ^3^Mean error between measured value and predictive equation (measured – predicted); ^4^Mean of absolute differences between measured and predictive value (Mean±SD); ^5^Percentage of subjects predicted by this predictive equation within ±10% of the measured value; ^6^Percentage of subjects predicted by this predictive equation <10% of the measured value; ^7^Percentage of subjects predicted by this predictive equation >10% of the measured value; ^8^Percentage of subjects predicted by this predictive equation within ±10% of the measured value; ^9^Percentage of subjects predicted by this predictive equation <10% of the measured value; ^10^Percentage of subjects predicted by this predictive equation >10% of the measured value. *P<0.05, **P<0.01, ***P<0.001, ANCOVA test. (a) and (b) refer to different predictive equations which are proposed by the same author but required different anthropometry or body composition parameters.

Supplementary material 2e: Validity of resting energy expenditure (REE) predictive equations in overweight young women.

| **REE predictive equation** | **N** | **^1^REE**  **(Kcal/day)** | **P value ANCOVA^2^** | **Mean BIAS^3^ (Kcal/day)** | **Lower limit of agreement (Kcal/day)** | **Higher limit of agreement (Kcal/day)** | **Mean absolute differences^4^ (Kcal/day)** | **Percentage of accurate predictions (10%)^5^** | **Percentage of under predictions (10%)^6^** | **Percentage of over predictions (10%)^7^** | **Percentage of accurate predictions (5%)^8^** | **Percentage of under predictions (5%)^9^** | **Percentage of over predictions (5%)^10^** |
| --- | --- | --- | --- | --- | --- | --- | --- | --- | --- | --- | --- | --- | --- |
| Harris & Benedict (1) | 21 | 1566±75 | 0.158 | -86 | -431 | 259 | 165±92 | 52.4 | 9.5 | 38.1 | 14.3 | 23.8 | 61.9 |
| Roza et al. (2) | 21 | 1578±80 | 0.173 | -98 | -445 | 249 | 171±96 | 52.4 | 9.5 | 38.1 | 19 | 19 | 61.9 |
| Bernstein et al. (3)a | 21 | 1265±48 | 0.139 | 216 | -126 | 557 | 227±154 | 33.3 | 66.7 | 0 | 19 | 76.2 | 4.8 |
| Bernstein et al. (3)b | 21 | 1097±82 | 0.139 | 384 | 74 | 693 | 383±154 | 0 | 100 | 0 | 0 | 100 | 0 |
| Owen et al. (4)a | 21 | 1330±47 | 0.142 | 150 | -193 | 493 | 187±126 | 42.9 | 52.4 | 4.8 | 14.3 | 71.4 | 14.3 |
| Owen et al. (4)b | 21 | 1137±78 | 0.143 | 343 | 44 | 642 | 342±149 | 14.3 | 85.7 | 0 | 0 | 100 | 0 |
| Mifflin et al. (5)a | 21 | 1499±103 | 0.218 | -19 | -378 | 340 | 152±89 | 52.4 | 23.8 | 23.8 | 19 | 33.3 | 47.6 |
| Mifflin et al. (5)b | 21 | 1216±78 | 0.143 | 264 | -35 | 563 | 263±149 | 19 | 81 | 0 | 14.3 | 85.7 | 0 |
| Livingston et al. (6) | 21 | 1505±65 | 0.141 | -25 | -368 | 317 | 145±88 | 52.4 | 19 | 28.6 | 28.6 | 28.6 | 42.9 |
| Schofield et al. (7)a | 21 | 1591±96 | 0.134 | -110 | -465 | 244 | 178±103 | 42.9 | 9.5 | 47.6 | 14.3 | 19 | 66.7 |
| Schofield et al. (7)a | 21 | 1564±103 | 0.165 | -84 | -443 | 275 | 171±93 | 52.4 | 14.3 | 33.3 | 9.5 | 23.8 | 66.7 |
| FAO (8)a | 21 | 1592±96 | 0.134 | -112 | -466 | 242 | 178±104 | 42.9 | 9.5 | 47.6 | 14.3 | 19 | 66.7 |
| FAO (8)a | 21 | 1575±105 | 0.173 | -95 | -455 | 265 | 175±97 | 52.4 | 9.5 | 38.1 | 9.5 | 23.8 | 66.7 |
| Henry et al. (9)a | 21 | 1529±85 | 0.133 | -49 | -398 | 301 | 153±91 | 57.1 | 14.3 | 28.6 | 23.8 | 28.6 | 47.6 |
| Henry et al. (9)b | 21 | 1498±101 | 0.209 | -18 | -377 | 342 | 149±95 | 52.4 | 23.8 | 23.8 | 28.6 | 33.3 | 38.1 |
| Muller et al. (10)a | 21 | 1546±196 | 0.552 | -66 | -479 | 348 | 173±124 | 52.4 | 14.3 | 33.3 | 23.8 | 23.8 | 52.4 |
| Muller et al. (10)b | 21 | 1496±175 | 0.550 | -16 | -431 | 399 | 164±122 | 42.9 | 23.8 | 33.3 | 28.6 | 28.6 | 42.9 |
| Korth et al. (11)a | 21 | 1596±115 | 0.252 | -116 | -483 | 252 | 188±102 | 33.3 | 14.3 | 52.4 | 14.3 | 19 | 66.7 |
| Korth et al. (11)b | 21 | 1347±103 | 0.150 | 133 | -163 | 429 | 158±119 | 61.9 | 38.1 | 0 | 23.8 | 61.9 | 14.3 |
| De Lorenzo et al. (12) | 21 | 1578±95 | 0.181 | -98 | -452 | 256 | 175±94 | 47.6 | 9.5 | 42.9 | 9.5 | 23.8 | 66.7 |
| Johnstone et al. (13) | 21 | 1440±100 | 0.142 | 40 | -281 | 361 | 126±103 | 57.1 | 28.6 | 14.3 | 42.9 | 33.3 | 23.8 |
| Weijs & Vansant (18) | 21 | 1680±116 | 0.192 | -199 | -567 | 168 | 228±144 | 33.3 | 0 | 66.7 | 19 | 9.5 | 71.4 |
| Frankenfield (14)a | 21 | 1579±85 | 0.172 | -99 | -448 | 250 | 173±95 | 42.9 | 9.5 | 47.6 | 14.3 | 19 | 66.7 |
| Frankenfield (14)b | 21 | 1448±69 | 0.140 | 33 | -310 | 375 | 138±102 | 52.4 | 28.6 | 19 | 38.1 | 33.3 | 28.6 |
| De la Cruz et al. (15) | 21 | 1758±216 | 0.531 | -277 | -725 | 170 | 303±183 | 19 | 4.8 | 76.2 | 19 | 4.8 | 76.2 |
| Willis et al. (16) | 21 | 1452±78 | 0.140 | 28 | -317 | 373 | 137±103 | 52.4 | 28.6 | 19 | 38.1 | 33.3 | 28.6 |

^1^REE obtained by predictive equations (Mean±SD); ^2^P value of the main effect of ANCOVA comparing measured and predicted REE adjusting for the metabolic cart used; ^3^Mean error between measured value and predictive equation (measured – predicted); ^4^Mean of absolute differences between measured and predictive value (Mean±SD); ^5^Percentage of subjects predicted by this predictive equation within ±10% of the measured value; ^6^Percentage of subjects predicted by this predictive equation <10% of the measured value; ^7^Percentage of subjects predicted by this predictive equation >10% of the measured value; ^8^Percentage of subjects predicted by this predictive equation within ±10% of the measured value; ^9^Percentage of subjects predicted by this predictive equation <10% of the measured value; ^10^Percentage of subjects predicted by this predictive equation >10% of the measured value. *P<0.05, **P<0.01, ***P<0.001, ANCOVA test. (a) and (b) refer to different predictive equations which are proposed by the same author but required different anthropometry or body composition parameters.

Table S2f: Validity of resting energy expenditure (REE) predictive equations in obese young women.

| **REE predictive equation** | **N** | **^1^REE**  **(Kcal/day)** | **P value ANCOVA^2^** | **Mean BIAS^3^ (Kcal/day)** | **Lower limit of agreement (Kcal/day)** | **Higher limit of agreement (Kcal/day)** | **Mean absolute differences^4^ (Kcal/day)** | **Percentage of accurate predictions (10%)^5^** | **Percentage of under predictions (10%)^6^** | **Percentage of over predictions (10%)^7^** | **Percentage of accurate predictions (5%)^8^** | **Percentage of under predictions (5%)^9^** | **Percentage of over predictions (5%)^10^** |
| --- | --- | --- | --- | --- | --- | --- | --- | --- | --- | --- | --- | --- | --- |
| Harris & Benedict (1) | 9 | 1665±108 | 0.451 | -196 | -617 | 225 | 245±139 | 11.1 | 11.1 | 77.8 | 11.1 | 11.1 | 77.8 |
| Roza et al. (2) | 9 | 1673±115 | 0.466 | -204 | -635 | 227 | 251±147 | 11.1 | 11.1 | 77.8 | 11.1 | 11.1 | 77.8 |
| Bernstein et al. (3)a | 9 | 1342±69 | 0.345 | 127 | -268 | 523 | 162±166 | 55.6 | 33.3 | 11.1 | 44.4 | 44.4 | 11.1 |
| Bernstein et al. (3)b | 9 | 1191±126 | 0.385 | 278 | -164 | 721 | 295±193 | 33.3 | 66.7 | 0 | 0 | 88.9 | 11.1 |
| Owen et al. (4)a | 9 | 1401±69 | 0.339 | 68 | -333 | 470 | 136±156 | 66.7 | 22.2 | 11.1 | 55.6 | 33.3 | 11.1 |
| Owen et al. (4)b | 9 | 1210±119 | 0.346 | 259 | -186 | 704 | 276±197 | 33.3 | 66.7 | 0 | 0 | 88.9 | 11.1 |
| Mifflin et al. (5)a | 9 | 1601±149 | 0.553 | -132 | -601 | 336 | 213±152 | 44.4 | 11.1 | 44.4 | 11.1 | 11.1 | 77.8 |
| Mifflin et al. (5)b | 9 | 1289±119 | 0.346 | 180 | -265 | 625 | 215±183 | 44.4 | 44.4 | 11.1 | 22.2 | 66.7 | 11.1 |
| Livingston et al. (6) | 9 | 1600±85 | 0.384 | -131 | -531 | 269 | 209±100 | 22.2 | 11.1 | 66.7 | 0 | 22.2 | 77.8 |
| Schofield et al. (7)a | 9 | 1737±143 | 0.566 | -268 | -715 | 180 | 303±164 | 22.2 | 0 | 77.8 | 0 | 11.1 | 88.9 |
| Schofield et al. (7)a | 9 | 1697±154 | 0.582 | -228 | -695 | 239 | 275±166 | 11.1 | 11.1 | 77.8 | 11.1 | 11.1 | 77.8 |
| FAO (8)a | 9 | 1737±142 | 0.563 | -268 | -714 | 178 | 303±164 | 22.2 | 0 | 77.8 | 0 | 11.1 | 88.9 |
| FAO (8)a | 9 | 1706±156 | 0.584 | -237 | -708 | 234 | 282±169 | 11.1 | 11.1 | 77.8 | 11.1 | 11.1 | 77.8 |
| Henry et al. (9)a | 9 | 1658±126 | 0.510 | -189 | -621 | 244 | 238±150 | 22.2 | 11.1 | 66.7 | 11.1 | 11.1 | 77.8 |
| Henry et al. (9)b | 9 | 1598±150 | 0.547 | -129 | -605 | 346 | 213±155 | 44.4 | 11.1 | 44.4 | 11.1 | 11.1 | 77.8 |
| Muller et al. (10)a | 9 | 1575±150 | 0.919 | -106 | -552 | 340 | 193±142 | 44.4 | 11.1 | 44.4 | 22.2 | 22.2 | 55.6 |
| Muller et al. (10)b | 9 | 1512±160 | 0.891 | -43 | -493 | 406 | 176±132 | 44.4 | 22.2 | 33.3 | 22.2 | 33.3 | 44.4 |
| Korth et al. (11)a | 9 | 1696±165 | 0.589 | -227 | -720 | 266 | 276±181 | 11.1 | 11.1 | 77.8 | 11.1 | 11.1 | 77.8 |
| Korth et al. (11)b | 9 | 1442±156 | 0.416 | 27 | -454 | 508 | 192±130 | 33.3 | 33.3 | 33.3 | 22.2 | 44.4 | 33.3 |
| Lazzer et al. (17) | 9 | 1690±138 | 0.534 | -221 | -673 | 230 | 266±162 | 11.1 | 11.1 | 77.8 | 11.1 | 11.1 | 77.8 |
| Johnstone et al. (13) | 9 | 1169±159 | 0.093 | 300 | -226 | 827 | 300±263 | 44.4 | 55.6 | 0 | 11.1 | 88.9 | 0 |
| Weijs & Vansant (18) | 9 | 1568±154 | 0.469 | -99 | -562 | 364 | 186±159 | 44.4 | 11.1 | 44.4 | 33.3 | 11.1 | 55.6 |
| Frankenfield (14)a | 9 | 1818±172 | 0.631 | -349 | -840 | 142 | 372±202 | 11.1 | 0 | 88.9 | 0 | 11.1 | 88.9 |
| Frankenfield (14)b | 9 | 1668±122 | 0.489 | -199 | -634 | 235 | 248±150 | 11.1 | 11.1 | 77.8 | 11.1 | 11.1 | 77.8 |
| De la Cruz et al. (15) | 9 | 1601±97 | 0.428 | -132 | -539 | 275 | 208±110 | 22.2 | 11.1 | 66.7 | 11.1 | 11.1 | 77.8 |
| Willis et al. (16) | 9 | 1725±204 | 0.648 | -256 | -765 | 253 | 268±239 | 44.4 | 0 | 55.6 | 33.3 | 0 | 66.7 |
| De Luis et al. (18) | 9 | 1822±92 | 0.151 | -352 | -846 | 137 | 357±190 | 22.2 | 0 | 77.8 | 11.1 | 0 | 88.9 |

^1^REE obtained by predictive equations (Mean±SD); ^2^P value of the main effect of ANCOVA comparing measured and predicted REE adjusting for the metabolic cart used; ^3^Mean error between measured value and predictive equation (measured – predicted); ^4^Mean of absolute differences between measured and predictive value (Mean±SD); ^5^Percentage of subjects predicted by this predictive equation within ±10% of the measured value; ^6^Percentage of subjects predicted by this predictive equation <10% of the measured value; ^7^Percentage of subjects predicted by this predictive equation >10% of the measured value; ^8^Percentage of subjects predicted by this predictive equation within ±10% of the measured value; ^9^Percentage of subjects predicted by this predictive equation <10% of the measured value; ^10^Percentage of subjects predicted by this predictive equation >10% of the measured value. *P<0.05, **P<0.01, ***P<0.001, ANCOVA test. (a) and (b) refer to different predictive equations which are proposed by the same author but required different anthropometry or body composition parameters.

**REFERENCES**

1. Harris J, Benedict F. A biometric study of basal metabolism in man. Proc Natl Acad Sci. 1918;4(12):370–3.

2. Roza AM, Shizgal HM. The Harris Benedict equation reevaluated: resting energy requirements and the body cell mass. Am J Clin Nutr. 1984;40(1):168–82.

3. Bernstein RS, Thornton JC, Yang MU, Wang J, Redmond AM, Pierson RN, et al. Prediction of the resting metabolic rate in obese patients. Am J Clin Nutr. 1983;37(4):595–602.

4. Owen OE, Kavle E, Owen RS, Polansky M, Caprio S, Mozzoli MA, et al. A reappraisal of caloric requirements in healthy women. Am J Clin Nutr. 1986;44(1):1–19.

5. Mifflin MD, St Jeor ST, Hill LA, Scott BJ, Daugherty SA, Koh YO. A new predictive equation for resting energy expenditure in healthy individuals. Am J Clin Nutr. 1990;51(2):241–7.

6. Livingston EH, Kohlstadt I. Simplified resting metabolic rate-predicting formulas for normal-sized and obese individuals. Obes Res. 2005;13(7):1255–62.

7. Schofield WN. Predicting basal metabolic rate, new standards and review of previous work. Hum Nutr Clin Nutr. 1985;39 Suppl 1:5–41.

8. FAO/WHO/UNU. Energy and protein requirements. Geneva, Switz World Heal Organ Tech Rep Ser. 1985;

9. Henry CJK. Basal metabolic rate studies in humans: measurement and development of new equations. Public Health Nutr. 2005;8(7A):1133–52.

10. Müller MJ, Bosy-Westphal A, Klaus S, Kreymann G, Lührmann PM, Neuhäuser-Berthold M, et al. World Health Organization equations have shortcomings for predicting resting energy expenditure in persons from a modern, affluent population: generation of a new reference standard from a retrospective analysis of a German database of resting energy expe. Am J Clin Nutr. 2004;80(5):1379–90.

11. Korth O, Bosy-Westphal A, Zschoche P, Glüer CC, Heller M, Müller MJ. Influence of methods used in body composition analysis on the prediction of resting energy expenditure. Eur J Clin Nutr. 2007;61(5):582–9.

12. De Lorenzo A, Tagliabue A, Andreoli A, Testolin G, Comelli M, Deurenberg P. Measured and predicted resting metabolic rate in Italian males and females, aged 18-59 y. Eur J Clin Nutr. 2001;55(3):208–14.

13. Johnstone AM, Rance KA, Murison SD, Duncan JS, Speakman JR. Additional anthropometric measures may improve the predictability of basal metabolic rate in adult subjects. Eur J Clin Nutr. 2006;60(12):1437–44.

14. Frankenfield DC. Bias and accuracy of resting metabolic rate equations in non-obese and obese adults. Clin Nutr. 2013;32(6):976–82.

15. de la Cruz Marcos S, de Mateo Silleras B, Camina Martín MA, Carreño Enciso L, Miján de la Torre A, Galgani Fuentes JE, et al. Proposal for a new formula for estimating resting energy expenditure for healthy spanish population. Nutr Hosp. 2015;32(5):2346–52.

16. Willis EA, Herrmann SD, Ptomey LT, Honas JJ, Bessmer CT, Donnelly JE, et al. Predicting resting energy expenditure in young adults. Obes Res Clin Pract. 2014;8(3):201–8.

17. Lazzer S, Agosti F, Resnik M, Marazzi N, Mornati D, Sartorio A. Prediction of resting energy expenditure in severely obese Italian males. J Endocrinol Invest. 2007;30(9):754–61.

18. Weijs PJM, Vansant GAAM. Validity of predictive equations for resting energy expenditure in Belgian normal weight to morbid obese women. Clin Nutr. 2010;29(3):347–51.
